# Validation of a Thin-Layer Chromatography for the Determination of Hydrocortisone Acetate and Lidocaine in a Pharmaceutical Preparation

**DOI:** 10.1155/2014/107879

**Published:** 2014-01-06

**Authors:** Małgorzata Dołowy, Katarzyna Kulpińska-Kucia, Alina Pyka

**Affiliations:** Department of Analytical Chemistry, Faculty of Pharmacy, Medical University of Silesia, PL-4 Jagiellońska Street, 41-200 Sosnowiec, Poland

## Abstract

A new specific, precise, accurate, and robust TLC-densitometry has been developed for the simultaneous determination of hydrocortisone acetate and lidocaine hydrochloride in combined pharmaceutical formulation. The chromatographic analysis was carried out using a mobile phase consisting of chloroform + acetone + ammonia (25%) in volume composition 8 : 2 : 0.1 and silica gel 60F_254_ plates. Densitometric detection was performed in UV at wavelengths 200 nm and 250 nm, respectively, for lidocaine hydrochloride and hydrocortisone acetate. The validation of the proposed method was performed in terms of specificity, linearity, limit of detection (LOD), limit of quantification (LOQ), precision, accuracy, and robustness. The applied TLC procedure is linear in hydrocortisone acetate concentration range of 3.75 ÷ 12.50 **μ**g*·*spot^−1^, and from 1.00 ÷ 2.50 **μ**g*·*spot^−1^ for lidocaine hydrochloride. The developed method was found to be accurate (the value of the coefficient of variation CV [%] is less than 3%), precise (CV [%] is less than 2%), specific, and robust. LOQ of hydrocortisone acetate is 0.198 **μ**g*·*spot^−1^ and LOD is 0.066 **μ**g*·*spot^−1^. LOQ and LOD values for lidocaine hydrochloride are 0.270 and 0.090 **μ**g*·*spot^−1^, respectively. The assay value of both bioactive substances is consistent with the limits recommended by Pharmacopoeia.

## 1. Introduction

Corticosteroids are a class of drugs used widely in medicine as an anti-inflammatory and anti-allergic agent. Hydrocortisone acetate is the member of corticosteroid group. This compound usually exists as an ingredient of simple pharmaceutical formulations or in combination with antimicrobial and local anesthetic agents in form of tablets, creams, and injection solutions, respectively. The injection dosage form of hydrocortisone acetate and lidocaine hydrochloride is used to treat many different conditions such as allergic and breathing disorders or skin conditions. Literature concerning quantitative analysis of hydrocortisone acetate in pharmaceutical formulations, especially in form of injection solutions, is relatively sparse. The first report on hydrocortisone acetate analysis in commercial products dated back to 1957, when hydrocortisone acetate in combination with chloramphenicol was simultaneously estimated by UV-spectrophotometric method [1]. In 1967, Furoda and Hashizume proposed X-ray diffractometric determination of hydrocortisone acetate in ointment, which consists of hydrocortisone acetate and some antibiotics such as chloramphenicol and tetracycline hydrochloride [2]. A review of current literature shows that among various analytical methods, the modern chromatographic techniques like high performance liquid chromatography in reversed phase system (RP-HPLC) and ultra-performance liquid chromatography (UPLC) are widely applied in analysis of hydrocortisone acetate in various pharmaceutical formulations in form of ointments, creams, and suppositories [3–5]. To our knowledge, only one TLC method has been reported for the estimation of hydrocortisone acetate in combined dosage forms. In 1977, Amin and Jakobs described a direct TLC quantitative analysis of hydrocortisone acetate from ointments and suppositories [6]. The methods recommended by Polish and United States Pharmacopoeias for hydrocortisone acetate determination in bulk drug are UV-spectrophotometry and HPLC-UV [7, 8].

Lidocaine hydrochloride (previously known as lignocaine) is local anesthetic amide with antiarrhythmic action. This compound is usually available in tablets and also in form of injectable medicaments used in surgical intervention and in dentistry practice. Literature data on the analysis of lidocaine hydrochloride concerns mostly its determination in biological fluids to control the concentration of lidocaine hydrochloride and its metabolites in human organism for purpose of therapeutic drug monitoring [9]. As it was reported in the literature, lidocaine has been studied in biological samples, for example, in the women plasma after childbirth, in neonatal plasma, in human blood and also in cerebrospinal fluids [9–12]. A thorough literature survey revealed that some high performance liquid chromatography coupled with UV detector and MS/MS spectrometer (LC-UV, LC-MS/MS) and also capillary electrophoresis are available for the estimation of lidocaine hydrochloride in biological fluids [9–11]. Another work indicated that lidocaine and its metabolites in urine samples can be successfully determined by GC-MS method [12]. Lidocaine can exist in simple and combined pharmaceutical formulations (e.g., with cetylpyridinium chloride, cetrimonium bromide, and diclofenac diethylamine or with epinephrine). In pharmaceutical formulations and alginate microspheres containing lidocaine, it has been determined using RP-HPLC with diode array detector (HPLC-DAD) [13, 14]. Lidocaine is an active substance described in Pharmacopoeia's monograph. According to Polish and United States Pharmacopoeias, the method recommended for lidocaine analysis in pharmaceutical formulations and in bulk drugs is liquid chromatography with UV detection and also potentiometric and acidimetric titration [7, 8].

An extensive literature review shows that there are only few reports concerning stability indicating method for simultaneous determination of both pharmacological active compounds: hydrocortisone acetate and lidocaine hydrochloride in combined dosage forms. Out of various analytical methods, the multivariate regression spectrophotometry, micellar capillary electrophoresis (MEKC), and also RP-HPLC coupled with electrochemical detector to quantitation of these substances in combined dosage forms were applied. However, all of those studies have focused almost on the hydrocortisone acetate and lidocaine hydrochloride analysis in form of suppositories and ointments [15–17]. Therefore, attempts were made in this work to develop a fast, sensitive, robust, and cost-effective TLC-densitometric method suitable for quantitative analysis of hydrocortisone acetate and lidocaine hydrochloride in the commercial (injection) dosage form.

This paper is continuation of our previous studies concerning validation of thin-layer chromatography combined with densitometry (TLC-densitometry) for the determination of different bioactive substances in simple and complex dosage forms of many pharmaceutical formulations. In our earlier works, we suggested that the proposed TLC-densitometry may be an alternative method to the modern high performance liquid chromatography in the quality control of above-mentioned substances and it can be applied when HPLC or GC is not affordable in laboratory [18–25]. Additionally, we have stated that the full validation procedure of developed TLC-densitometric method for determination of bioactive substances in pharmaceutical formulations according to validation guidelines [26–29] is necessary for ensuring the reliability of obtained results in comparison to data described by manufacturer.

The objective of this study was to develop the chromatographic conditions suitable for separation and simultaneous determination of hydrocortisone acetate and lidocaine hydrochloride in the presence of substances related to hydrocortisone acetate (hydrocortisone, prednisolone, and cortisone acetate) in combined dosage injection solution (market available) by the use of TLC-densitometry. Hence, the developed TLC-densitometric method was fully evaluated with regard to obligatory validation procedures designed for quality and quantity control of pharmaceutical preparations [26–29].

## 2. Materials and Methods

### 2.1. Chemicals

The following solvents: ethanol (96%), chloroform, ammonia (25%), toluene, and ethyl acetate were purchased from POCh (Gliwice, Poland). Acetone was procured from Chempur (Piekary Śląskie, Poland). All solvents used for the TLC-densitometric analysis were of analytical grade. Hydrocortisone acetate (Ph. Eur. Grade) produced by Fluka Chemicals (Milwaukee, USA), lidocaine hydrochloride (>99%, Sigma-Aldrich, St. Louis, MO, USA), and the substances related to hydrocortisone acetate such as prednisolone (>99%, Sigma-Aldrich, St. Louis, MO, USA), cortisone acetate (>99%, Sigma-Aldrich, St. Louis, MO, USA), and also hydrocortisone (>99%, Sigma-Aldrich, St. Louis, MO, USA) were used as reference substances in the preparation of standard solutions.

### 2.2. Preparation of Combine Standard Solution

The standard stock solutions containing hydrocortisone acetate (125 mg) and lidocaine hydrochloride (25 mg) were prepared in a volumetric flask by dissolving both reference standard substances in a mixture of acetone + ethanol (1 : 1, v/v) and diluted up to volume 50 mL with solvent mixture. Final concentration of obtained standard solution was 12.50 mg of hydrocortisone acetate per 5 mL and 2.50 mg of lidocaine hydrochloride per 5 mL. Next, nine working solutions were prepared by appropriate dilutions of prepared standard solution with the same solvent mixture in order to obtain a concentration of 10.00, 8.70, 7.50, 6.25, 5.00, 3.75, 2.50, 1.88, and 1.25 mg of hydrocortisone acetate in 5 mL and the 2.00, 1.75, 1.50, 1.25, 1.00, 0.75, 0.50, 0.38, and 0.25 mg of lidocaine hydrochloride, respectively, in 5 mL of solution.

### 2.3. Pharmaceutical Preparation

The commercial pharmaceutical preparation of the combination, 125 mg of hydrocortisone acetate and 25 mg lidocaine hydrochloride in 5 mL ampoule, was used. This market available product is used mainly for intraarticular injection. Additionally, each ampoule contained respective preservatives as antimicrobial agents.

### 2.4. Sample Preparation

An ampoule (5 mL) of the examined product was transferred directly to 50 mL volumetric flask, dissolved in acetone + ethanol mixture (1 : 1, v/v), and diluted up to the mark with the same solvents. By this way, a sample solution containing 12.50 mg of hydrocortisone acetate and 2.50 mg lidocaine hydrochloride in 5 mL was obtained. Next, by the use of appropriate dilutions of prepared sample solution, three solutions at concentration of hydrocortisone acetate 10.00, 7.50, and 5.00 mg in 5 mL and lidocaine hydrochloride 2.00, 1.50, and 1.00 mg in 5 mL of sample solution were obtained. 5 *μ*L of sample solution for the TLC-densitometric analysis and quantitative determination of examined substances, hydrocortisone acetate and lidocaine hydrochloride, was used in each case.

### 2.5. Apparatus and Chromatographic Conditions

A thin-layer chromatography in normal phase system (NP-TLC) was performed on 10 cm × 20 cm aluminum plates precoated with silica gel 60F_254_ from Merck KGaA (number 1.05570, Darmstadt, Germany). Before using, the plates were activated at 120°C for 30 minutes. Next, 5 *μ*L of sample was spotted by means of a Camag (Muttenz, Switzerland) micropipette (5 *μ*L). The plates were developed at room temperature (20°C) in horizontal chamber from Camag 20 cm × 20 cm (number 0.222.5255, Muttenz, Switzerland) with the use of chloroform + acetone + ammonia (25%) in volume composition 8 : 2 : 0.1. The chamber was previously saturated with vapors of mobile phase for 30 minutes. The developing distance was 7.5 cm. Finally, the plates were dried for 20 h at 20°C in a fume cupboard. Additionally, a twin-trough chamber produced by Camag 20 cm × 10 cm (number 0.222.5221, Muttenz, Switzerland) and silica gel 60F_254_ (number 1.05554, Darmstadt, Germany) was used to robust test. Densitometric and spectrodensitometric analysis was carried out by densitometer (Camag, Muttenz, Switzerland) operated in absorbance mode and equipped with TLC Scanner 3. All the parameters were controlled by WinCATS 1.4.2 software. Deuterium lamp was used as a source of radiation. After spectrodensitometric analysis, densitometric determination of hydrocortisone acetate was made at absorption maximum equal to 250 nm and for lidocaine hydrochloride at a wavelength 200 nm.

### 2.6. Method Validation

The proposed method was validated in terms of range, specificity, linearity, precision, accuracy, quantification limit (LOQ), detection limit (LOD), and robustness in accordance with established International Conference on Harmonization (ICH) guideline [26] and validation guides published by Ferenczi-Fodor et al. [27, 28] and also by Nagy-Turák et al. [29].

#### 2.6.1. Specificity

Specificity of the method was checked by TLC-chromatography of working standard solutions of hydrocortisone acetate, lidocaine hydrochloride, and substances related to hydrocortisone acetate (prednisolone, hydrocortisone, and cortisone acetate) and sample solution containing hydrocortisone acetate and lidocaine hydrochloride obtained from the commercial product. In order to determine the specificity, appropriate chromatographic conditions were applied (such as suitable adsorbent and a proper mobile phase), which allowed the complete separation of hydrocortisone acetate and lidocaine hydrochloride in the presence of substances related to hydrocortisone acetate, mentioned above. To estimate the specificity of developed method, comparison of obtained chromatographic bands was made.

Moreover, the effect of resolution of both examined bioactive substances and the other ones presented in the examined sample, including the substances related to hydrocortisone acetate and preservatives, the following separation factor for active constituents was calculated [30]:

(1) $$\begin{array}{c} R_{S}=\frac{2d}{w_{b1}+w_{b2}}, \end{array}$$

where *d* is distance between the centers of two adjacent chromatographic bands *w*_*b*1_ and *w*_*b*2_ that are band width at base.

#### 2.6.2. Linearity

Linearity test of developed TLC-densitometric method was made by the dilution of the reference standard solutions of hydrocortisone acetate and lidocaine hydrochloride to the required concentration. For this aim, a series of nine solutions containing 10.00, 8.70, 7.50, 6.25, 5.00, 3.75, 2.50, 1.88, and 1.25 mg of hydrocortisone acetate in 5 mL of solution and 2.00, 1.75, 1.50, 1.25, 1.00, 0.75, 0.50, 0.38, and 0.25 mg of lidocaine hydrochloride in 5 mL of solution were prepared. Five *μ*L of each solution was spotted on previously activated TLC plate. The plates were developed using a mobile phase consisting of chloroform + acetone + ammonia (25%) in volume composition 8 : 2 : 0.1 and next scanned. This analysis was repeated three times. Calibration curve was plotted between peak area *A* [AU] versus concentration of examined substances *x*  [*μ*g · spot^−1^]. The coefficient correlation, slope, and intercept of obtained calibration plots were reported.

#### 2.6.3. Limit of Detection (LOD) and Quantification (LOQ)

The LOD and LOQ values were determined on the basis of specific calibration curve using the samples containing both reference standards at respective concentrations:

- 0.50, 0.40, and 0.30 mg of hydrocortisone acetate in 5 mL of solution,
- 0.75, 0.50, and 0.38 mg of lidocaine hydrochloride in 5 mL of solution.

Five *μ*L of prepared solutions was used in this analysis. The results are the means of three measurements.

The detection limit (LOD) was calculated as

(2) $$\begin{array}{c} \text{LOD}=\frac{3.3\sigma }{S}. \end{array}$$

The quantification limit (LOQ) was calculated as

(3) $$\begin{array}{c} \text{LOQ}=\frac{10\sigma }{S}, \end{array}$$

where *σ* = the standard deviation of the response and *S* = the slope of the calibration curve.

#### 2.6.4. Accuracy

The accuracy of the method was evaluated by measurement of recovery. Percent recovery was performed by the standard addition method. For this aim, known amounts of reference standard hydrocortisone acetate and lidocaine hydrochloride were added to the sample in given quantity: 50%, 100%, and 150% level of test concentration (10.00, 7.50, and 5.00 per 5 mL for hydrocortisone acetate) and (2.00, 1.50, and 1.00 per 5 mL for lidocaine hydrochloride). This analysis was performed six times. The percentage of recovery for both drug components was calculated.

#### 2.6.5. Precision

Repeatability (intraday precision) of the method was determined by the analysis of three replicates of sample solutions at three different concentrations of hydrocortisone acetate (10.00, 7.50, and 5.00 per 5 mL of solution) and for lidocaine hydrochloride (2.00, 1.50, and 1.00 per 5 mL of solution). All solutions were prepared independently and repeated three times. 5 *μ*L of prepared solutions was used in each case. Precision was determined on the basis of densitometric measurements of obtained spots as the relative standard deviation (coefficient of variation: CV [%]).

#### 2.6.6. Robustness

The robustness of the method was evaluated during its development by making changes to the method parameters. Robustness test was prepared according to guidelines described in the papers by Ferenczi-Fodor et al. [27, 28] and Nagy-Turák et al. [29]. 7.50 and 1.50 *μ*g·spot^−1^ of hydrocortisone acetate and lidocaine hydrochloride, respectively, were spotted on the plates and next the plates were developed after altering the conditions. The conditions changed were the sorbent type (number 1.05570, number 1.05554), the chamber type (20 cm × 10 cm, 20 cm × 20 cm), the temperature of plate activation (±10°C), the distance of development (±0.5 cm), saturation time of the chamber (±2 min), the volume of chloroform (±0.1 mL), and the volume of acetone (±0.1 mL) in used mobile phase. The main effects of seven factors were tested on two levels in eight experiments [27–29]. The levels of factors investigated and the experimental design matrix (2^3^) are shown in Table 1. A high level is represented by “+” and a low level by “−.” The ways of calculation of the effects (*E*) characterizing the particular individual factors and rank probabilities were earlier presented [18–23].

## 3. Results and Discussion

### 3.1. Optimization of TLC Method

In our previous paper [19], we confirmed that normal phase thin-layer chromatography (NP-TLC) combined with densitometry is a method which can be successfully applied for identification and quantification of hydrocortisone in a simple pharmaceutical formulation in form of tablets containing 20 mg of hydrocortisone per tablet. We found that the optimal separation of hydrocortisone from its related substances: hydrocortisone acetate and prednisolone can be achieved on silica gel 60F_254_ and by the use of mobile phase: acetone + *n*-hexane + glacial acetic acid in volume composition 5 mL: 5 mL: 0.05 mL. Based on these results, elaboration of the TLC-densitometric method for quantitation of hydrocortisone acetate in combined with lidocaine hydrochloride injection solution (ampoule 5 mL) was begun from optimization of the chromatographic conditions, necessary for simultaneous determination of hydrocortisone acetate and lidocaine hydrochloride in this solution. Therefore, besides the above mentioned mixture, the new two mobile phases were applied to develop the chromatographic plates for analysis of sample solution and the reference standard solutions of drug components. The first mobile phase used in experiment was a mixture of the following solvents: chloroform + acetone + ammonia (25%) in volume composition 8 : 2 : 0.1 and the second mobile phase used was a mixture of toluene + ethyl acetate in volume composition 15 : 35. Finally, the obtained results indicated that the optimum mobile phase for separation of lidocaine hydrochloride, hydrocortisone acetate, and also the substances related to hydrocortisone acetate, for example, hydrocortisone, prednisolone, and cortisone acetate, is the first mobile phase. Application of chloroform + acetone + ammonia (25%) as a mobile phase resulted in obtaining compact bands and symmetric peaks for both examined substances. Additionally, a confirmation of the presence of preservatives in the sample of studied drug was possible. Thus, it can be suggested that the optimized NP-TLC technique is suitable for quantitative determination of hydrocortisone acetate and lidocaine hydrochloride in combined preparations in form of injectable solution.

#### 3.1.1. Method Validation

**Figure 1:**
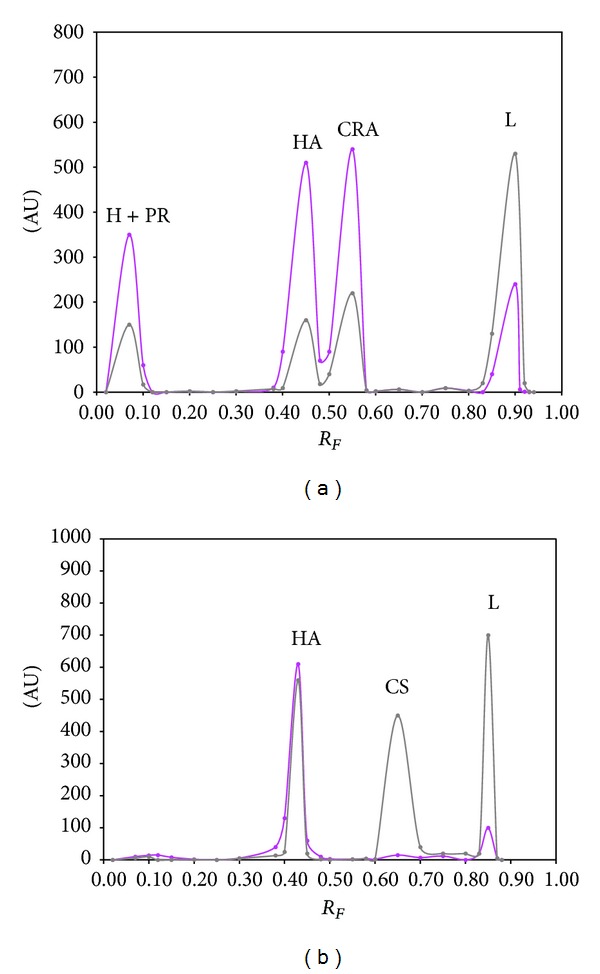
(a) Densitogram of the combine standard solution containing the following reference substances: lidocaine hydrochloride (L), hydrocortisone acetate (HA) and its related substances, namely, hydrocortisone (H), prednisolone (PR), and cortisone acetate (CRA) and (b) densitogram of drug sample analyzed by NP-TLC, at wavelengths of 200 nm and 250 nm obtained under optimum chromatographic conditions.

**Figure 2:**
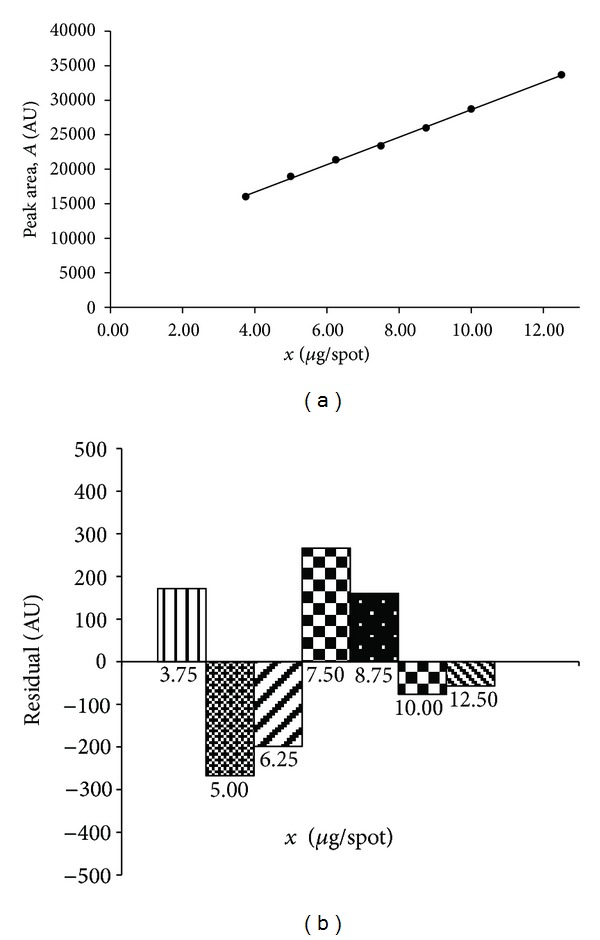
Calibration plot for hydrocortisone acetate (a) and plot of residuals (b) for hydrocortisone acetate in linear working range.

**Figure 3:**
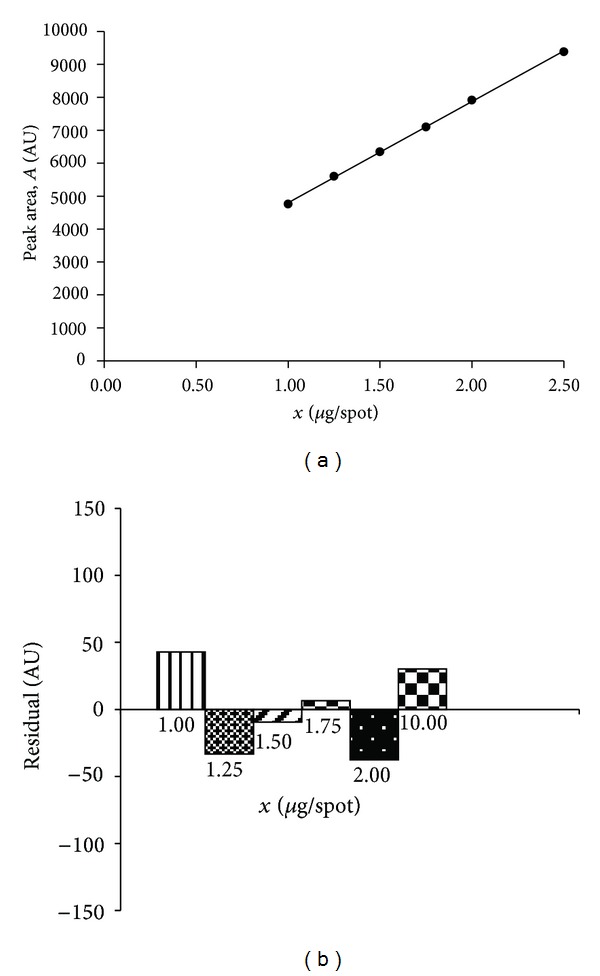
Calibration plot for lidocaine hydrochloride (a) and plot of residuals (b) for lidocaine hydrochloride in linear working range.

**Figure 4:**
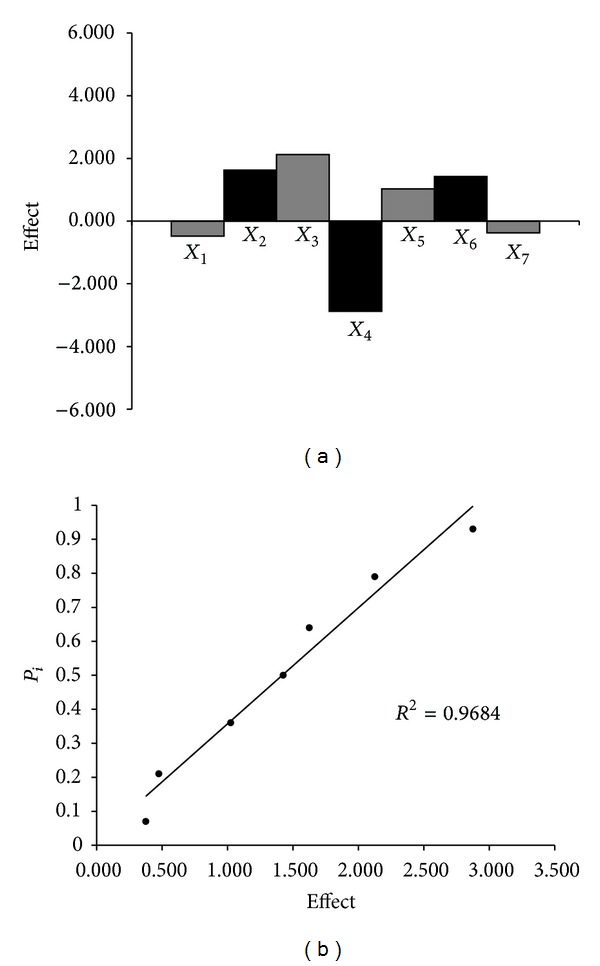
Robustness test: the effect of the factors (a) and a half-normal probability plot of effects (b) for determination of hydrocortisone acetate in examined drug.

**Figure 5:**
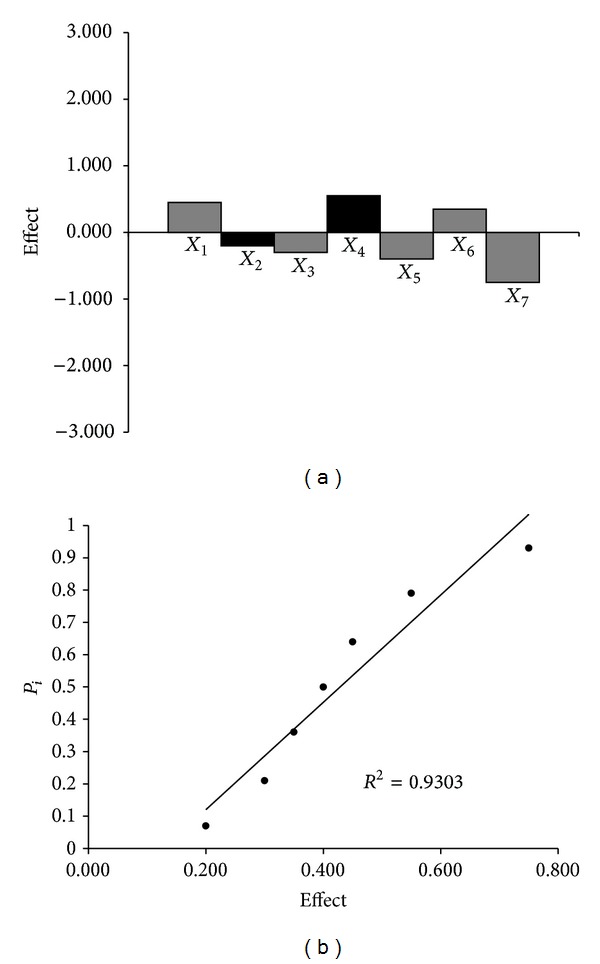
Robustness test: the effect of the factors (a) and half-normal probability plot of effects (b) for determination of lidocaine hydrochloride in examined drug.

Summarized results of the method validation are presented in Figures 1–5 as well as in Tables 1 and 2.

#### 3.1.2. Specificity

In order to verify the specificity of applied TLC-densitometric method, the mixture of chloroform + acetone + ammonia (25%) in volume composition 8 : 2 : 0.1 was used. As we described above, this mixture was chosen for development as the optimum mobile, because it enabled a successful separation of hydrocortisone acetate (HA) from lidocaine hydrochloride (L) and from its related substances, namely, prednisolone (PR), cortisone acetate (CRA), and hydrocortisone (H) presented in commercial injection solution. Typical densitogram obtained from combine standard solution under these chromatographic conditions is performed in Figure 1(a).

On the basis of Figure 1(a), it can be observed that the hydrocortisone (H) and prednisolone (PR) form one compact band with *R*_*F*_ value 0.07 ± 0.01. The *R*_*F*_ values of hydrocortisone acetate (HA), cortisone acetate (CRA), and lidocaine hydrochloride (L) are as follows: *R*_*F*(HA)_ = 0.41 ± 0.02, *R*_*F*(CRA)_ = 0.55 ± 0.02, and *R*_*F*(L)_ = 0.85 ± 0.03. While the rate of *R*_*S*_ (calculated accordance to (1)) received the following: *R*_*S*(H,PR/HA)_ = 3.30, *R*_*S*(HA/CRA)_ = 1.17, and *R*_*S*(CRA/L)_ = 3.43. Densitometric scan of obtained bands was made at the maximum absorption (*λ*_max_) for each substance. In the case of hydrocortisone, prednisolone, and hydrocortisone acetate, the wavelength 250 nm was used, for cortisone acetate 247 nm, and for lidocaine hydrochloride 200 nm. The high selectivity of the adsorption TLC combined with densitometry for the quantification of hydrocortisone acetate and lidocaine hydrochloride in the pharmaceutical preparation in form of injection solution confirms densitogram of lidocaine hydrochloride (L) and hydrocortisone acetate (HA) obtained from examined drug, which was shown in Figure 1(b). No interference from substances related to hydrocortisone acetate and other components (e.g., preservatives) and contamination was observed. Moreover, additional components like preservatives (CS) present in the examined drug were well separated from hydrocortisone acetate and lidocaine hydrochloride, and it did not affect the TLC-densitometric analysis of HA and L. The value of *R*_*F*(CS)_ is 0.64 ± 0.02, while the main band of CS occurs at 200 nm. The average *R*_*F*_ values of hydrocortisone acetate and lidocaine hydrochloride were 0.41 and 0.84, respectively. The *R*_*F*_ values of hydrocortisone acetate and lidocaine hydrochloride, which were determined in the examined drug, are compatible with the *R*_*F*_ values of reference standard hydrocortisone acetate and lidocaine hydrochloride. Moreover, the specificity of developed method confirms the spectrodensitograms of examined substances: lidocaine hydrochloride and hydrocortisone acetate obtained from commercial product, which are identical with spectrodensitograms of reference standards of lidocaine hydrochloride and hydrocortisone acetate.

#### 3.1.3. Accuracy

Accuracy of the TLC-densitometric method was evaluated by the measurement of recovery. Recovery of hydrocortisone acetate in the examined drug ranged from 95.4% to 101.4%, from 97.1% to 101.4%, and from 95.1% to 101.4%, respectively, for 50%, 100%, and 150% amount of standard hydrocortisone acetate added to the pharmaceutical preparation. Generally, the average recovery was equal to 97.6%, 99.0%, and 98.0%. Recovery of lidocaine hydrochloride coming from the same pharmaceutical preparation (injection solution) ranged from 95.3% to 100.3%, from 95.3% to 98.7%, and from 97.2% to 101.6% for, respectively, 50%, 100%, and 150% standard lidocaine hydrochloride added to a solution of pharmaceutical formulation. An average recovery was equal to 97.6%, 97.0%, and 99.3% (Table 2). The values of the coefficient of variation less than 3% confirm the accuracy of the proposed method for the quantitative determination of hydrocortisone acetate and lidocaine hydrochloride in the combined pharmaceutical preparation.

#### 3.1.4. Linearity

Linearity of the proposed TLC method was determined by plotting peak area (*A*) [AU] versus concentration (*x*) of the proper standard substance: hydrocortisone acetate or lidocaine hydrochloride respectively, in *μ*g·spot^−1^ (Table 2). The plot (*n* = 7) was linear for hydrocortisone acetate in the range from 3.75 *μ*g·spot^−1^ to 12.50 *μ*g·spot^−1^ (Figure 2(a)). The calibration curve (*n* = 6) for lidocaine hydrochloride was linear in the range from 1.00 *μ*g·spot^−1^ to 2.50 *μ*g·spot^−1^, as it is shown in Figure 3(a). For both linear relationships, the high values of the correlation coefficients were achieved (*r* > 0.999). The statistical data of obtained linear dependencies, which confirm excellent linearity of two calibration curves, are presented in Table 2. Moreover, in both cases, the plots of residuals against the concentration of hydrocortisone acetate or lidocaine hydrochloride, respectively, were prepared. Difference between the obtained values and those estimated using a proper calibration curve is small. The residuals were distributed above and below the zero residual line for both examined substances (Figures 2(b) and 3(b)).

#### 3.1.5. Precision

Precision of the proposed method was investigated by densitometric measurements of spots obtained on the basis of sample solutions prepared at three different concentrations of hydrocortisone acetate and lidocaine hydrochloride (listed in Section 2). The results of this experiment were expressed in CV [%] (coefficient of variation) presented in Table 2. The results of CV for hydrocortisone acetate and lidocaine hydrochloride in the examined drug ranged from 0.92% to 1.32% and from 0.97% to 1.69%, respectively. The values of the coefficients of variation were less than 2% in each case. This fact indicates the closeness of agreement between a series of measurements obtained and confirms precision of developed TLC-densitometric method.

#### 3.1.6. Quantification Limit (LOQ) and Detection Limit (LOD)

As it was described previously, the limits of detection and quantification of the developed method were determined on the basis of calibration plot. The limits of quantification and detection for hydrocortisone acetate are, respectively, LOQ = 0.198 *μ*g·spot^−1^ and LOD = 0.066 *μ*g·spot^−1^. The limits of quantification and detection for lidocaine hydrochloride are, respectively, LOQ = 0.270 *μ*g·spot^−1^ and LOD = 0.090 *μ*g·spot^−1^.

The values obtained for LOD and LOQ were indicative of high sensitivity of the method for both substances presented in examined injection solution.

#### 3.1.7. Robustness

As we previously described, the robustness of the TLC-densitometric procedure was evaluated during development by making small, but deliberate, changes to the method parameters. The main effects of seven factors were tested on two levels in eight experiments. Table 1 shows the results obtained for hydrocortisone acetate and lidocaine hydrochloride content (*y*_*i*_) in the sample of examined injectable solution. On the basis of these results, it can be concluded that no factor has significant effect on the results. Moreover, the results were also evaluated by half-normal probability plot of rank probabilities (*p*_*i*_) as a function of the absolute values of the main effects. The effects of these factors and half-normal probability plot of the effects for the determination of hydrocortisone acetate and lidocaine hydrochloride in examined product are presented in Figures 4 and 5, respectively. The points of all factors lie near in the straight line, which indicates that their effect is negligible (*r* ^2^ ≥ 0.9303). Thus, the presented TLC-densitometric method was found to be robust with respect to variability in applied chromatographic conditions, except the variation of the ammonia (25%) content. The content of ammonia should be constant.

#### 3.1.8. Application of the Developed TLC-Densitometric Method to Pharmaceutical Formulation

The commercial injection solution of the hydrocortisone acetate and lidocaine hydrochloride mixture was successfully analyzed by the use of proposed TLC-method. The results of densitometric analysis of hydrocortisone acetate in form of densitometric bands coming from sample in which declared amount is equal to 7.50 *μ*g·spot^−1^ and lidocaine hydrochloride is equal to 1.50 *μ*g·spot^−1^ are demonstrated in Table 3. The content of both substances in the examined product was determined on the basis of previously described linear equation of the calibration curves determined for hydrocortisone acetate and lidocaine hydrochloride, respectively (Table 2). Moreover, in Table 3, the full statistical analysis of obtained results was performed. Percentage content of hydrocortisone acetate in the examined drugin injection dosage form determined by developed TLC-densitometry is 95.0% in the relation to the amount declared by manufacturer. This fact shows that obtained hydrocortisone acetate amount complies with the United States Pharmacopoeia specification (monograph) for content of the hydrocortisone acetate in pharmaceutical formulations. According to the United States Pharmacopoeia, the recommended hydrocortisone acetate amount in pharmaceutical formulation administered via injection should be in the range from 90% to 110%.

Our results presented in Table 3 show that in the case of lidocaine hydrochloride, the content of this substance in the examined combined pharmaceutical preparation in comparison with the value given by manufacturer is 98.4%, that is consistent with that recommended by Polish and United States Pharmacopoeias. The lidocaine hydrochloride content in injection solutions according to both Pharmacopoeias should be in the range from 95% to 115% of the declared value.

Summarizing the results obtained in this study, it can be said that the validation process of an analytical method including the TLC-densitometric method developed in this work is a very powerful tool, necessary in quantitative determination of hydrocortisone acetate amount not only in simple pharmaceutical formulations but in combined dosage forms like, for example, injection solution too (5 mL ampoule). Because the validation report indicates that the developed method fulfills the validation criteria of an analytical method designated for quantity control of pharmaceutical formulations in terms of specificity, linearity, limit of detection, limit of quantification, precision, accuracy, and robustness, it can be suggested that the TLC-densitometric method, mentioned in this study, is suitable for the routine analysis of hydrocortisone acetate and lidocaine hydrochloride in quantity control laboratories. Moreover, the results of our TLC-method are comparable with those obtained by the use of RP-HPLC method [16]. Thus, it can be concluded that this method can be used as a substitute method for the accurate assay of the hydrocortisone acetate and lidocaine hydrochloride in combined formulations in form of injection solutions, for example, in situation when HPLC or GC is not affordable in laboratory.

## 4. Conclusions

The work highlights that an important advantage of developed TLC-densitometric method is that it can be successfully applied to determination of individual components of analyzed preparation such as hydrocortisone acetate and lidocaine hydrochloride, respectively, in combined dosage forms. The chromatographic plates precoated with silica gel 60F_254_ and a mixture of chloroform + acetone + ammonia (25%) in volume composition 8 : 2 : 0.1 as mobile phase resulted in compact bands and symmetric peaks of lidocaine hydrochloride and also hydrocortisone acetate. UV densitometry was performed in absorbance mode at 200 nm and 250 nm. Under these optimum conditions, complete separation of both bioactive substances and related substances to hydrocortisone acetate, namely, hydrocortisone, prednisolone, and cortisone acetate, are observed. The studies indicated that none of the excipients presented above interfered with the proposed assay method. This aspect of TLC-densitometric analysis is of great interest as it offers possibilities for the assay of hydrocortisone acetate and lidocaine hydrochloride in a combined dosage formulation like injection solution.

The validation performed in accordance with ICH guidelines and with Ferenczi-Fodor and Nagy-Turák papers of the developed TLC-densitometry in terms of specificity, range, linearity, accuracy, precision, detection limit, quantification limit, and robustness indicates that the elaborated method realizes the criterion of the linearity in required range of hydrocortisone acetate and lidocaine hydrochloride concentrations. The results of hydrocortisone acetate and lidocaine hydrochloride obtained from examined commercial product compared with the value given by manufacturer are consistent with those which are recommended by Polish and United States Pharmacopoeias. It could be said that the NP-TLC-densitometric method is a new suitable method for the routine analysis of hydrocortisone acetate and lidocaine hydrochloride in quantity control laboratories.

## Figures and Tables

**Table 1:** Experimental design matrix (2^3^) for robustness test for biological active substances coming from injection dosage form of examined drug.

Experiment no.	*X*_1_	*X*_2_	*X*_3_	*X*_4_	*X*_5_	*X*_6_	*X*_7_	Biological active substance^a^ content (*y*_*i*_) [mg] in examined drug	
								HA	L
1	+	+	+	+	+	+	+	123.7	24.8
2	+	+	−	+	−	−	−	119.5	25.9
3	+	−	+	−	−	+	−	124.3	25.6
4	+	−	−	−	+	−	+	121.4	24.4
5	−	+	+	−	+	−	−	126.0	24.2
6	−	+	−	−	−	+	+	123.9	24.5
7	−	−	+	+	−	−	+	120.1	24.6
8	−	−	−	+	+	+	−	120.8	25.6

Effect for HA, L in examined drug									
HA	−0.475	1.625	2.125	−2.875	1.025	1.425	−0.375		
L	0.450	−0.200	−0.300	0.550	−0.400	0.350	−0.750		

^a^HA: hydrocortisone acetate; L: lidocaine hydrochloride.

*X*_1_: sorbent type; *X*_2_: chamber type; *X*_3_: temperature of plate activation; *X*_4_: distance of development; *X*_5_: saturation time of the chamber; *X*_6_: volume of chloroform; *X*_7_: volume of acetone.

**Table 2:** Validation of the method. Data for the quantitative determination of hydrocortisone acetate and lidocaine hydrochloride by NP-TLC with densitometry^a^.

Method characteristic	Results Hydrocortisone acetate	Results Lidocaine hydrochloride
Specificity	Specific	Specific
Range [*μ*g·spot^−1^]	3.75 ÷ 12.50	1.00 ÷ 2.50
Linearity [*μ*g·spot^−1^]	*A* = 8721.6 (±245.4) + 1990.6 (±30.0) · *x* *n* = 7; *r* = 0.9994; *s* = 221.7; *F* = 4391.5; *P* < 0.0001	*A* = 1722.4 (±52.9) + 3075.5 (±30.4) · *x* *n* = 6; *r* = 0.9998; *s* = 36.7; *F* = 10223; *P* < 0.0001
Accuracy		
for 50% standard solutions added (*n* = 6)	*R* = 97.6% CV = 2.60%	*R* = 97.6% CV = 2.12%
for 100% standard solutions added (*n* = 6)	*R* = 99.0% CV = 1.62%	*R* = 97.0% CV = 1.44%
for 150% standard solutions added (*n* = 6)	*R* = 98.0% CV = 2.53%	*R* = 99.3% CV = 1.85%
Detection limit (LOD) [*μ*g·spot^−1^]	0.066	0.090
Quantification limit (LOQ) [*μ*g·spot^−1^]	0.198	0.270
Precision (CV [*% *])		
for 10.0 hydrocortisone acetate and 2.0 lidocaine hydrochloride *μ*g·spot^−1^ (*n* = 3)	0.93%	1.01%
for 7.5 hydrocortisone acetate and lidocaine hydrochloride 1.5 *μ*g·spot^−1^ (*n* = 3)	0.92%	1.69%
for 5.0 hydrocortisone acetate and 1.0 lidocaine hydrochloride *μ*g·spot^−1^ (*n* = 3)	1.32%	0.97%
Robustness (CV [%])	Robust	Robust

^a^ *A*: peak area [AU]; *x*: amount of analyzed drug [*μ*g·spot^−1^]; *r*: correlation coefficient; *R*: recovery [%]; CV: coefficient of variation [%].

**Table 3:** Statistical data of the results of quantitative determination of hydrocortisone acetate and lidocaine hydrochloride in injection dosage form of investigated commercial product by NP-TLC with densitometry.

Parameter	Hydrocortisone acetate	Lidocaine hydrochloride
Number of analyses	6	6
The declared amount of respective substance in one ampoule [mg]	125	25
The average value of content [mg]	118.7	24.6
Minimum [mg]	115.9	23.7
Maximum [mg]	120.9	24.9
Variance (*s* ^2^)	3.66	0.20
Standard deviation (SD)	1.9	0.4
Coefficient of variation (CV [%])	1.60	1.79
Confidence interval of the mean value at significance level 95%	*μ* = 118.7 ± 1.9	*μ* = 24.6 ± 0.4
Content [%] of bioactive substance calculated in relation to the value declared by manufacturer	95.0%	98.4%
